# MARCH2, a Novel Oncogene-regulated SNAIL E3 Ligase, Suppresses Triple-negative Breast Cancer Metastases

**DOI:** 10.1158/2767-9764.CRC-23-0090

**Published:** 2024-03-28

**Authors:** Koichi Ito, Ibuki Harada, Criseyda Martinez, Katsutoshi Sato, EunJee Lee, Elisa Port, Jessica H. Byerly, Anupma Nayak, Ekta Tripathi, Jun Zhu, Hanna Y. Irie

**Affiliations:** 1 Division of Hematology and Medical Oncology, Department of Medicine, Icahn School of Medicine at Mount Sinai, New York, New York.; 2 Sema4, Stamford, Connecticut.; 3 Department of Surgery, Mount Sinai Hospital, New York, New York.; 4 Department of Pathology and Laboratory Medicine, Perelman School of Medicine, University of Pennsylvania, Philadelphia, Pennsylvania.; 5 Department of Oncological Sciences, Tisch Cancer Institute, Icahn School of Medicine at Mount Sinai, New York, New York.

## Abstract

Epithelial–mesenchymal transition (EMT) in cancer promotes metastasis and chemotherapy resistance. A subset of triple-negative breast cancer (TNBC) exhibits a mesenchymal gene signature that is associated with poor patient outcomes. We previously identified PTK6 tyrosine kinase as an oncogenic driver of EMT in a subset of TNBC. PTK6 induces EMT by stabilizing SNAIL, a key EMT-initiating transcriptional factor. Inhibition of PTK6 activity reverses mesenchymal features of TNBC cells and suppresses their metastases by promoting SNAIL degradation via a novel mechanism. In the current study, we identify membrane-associated RING-CH2 (MARCH2) as a novel PTK6-regulated E3 ligase that promotes the ubiquitination and degradation of SNAIL protein. The MARCH2 RING domain is critical for SNAIL ubiquitination and subsequent degradation. PTK6 inhibition promotes the interaction of MARCH2 with SNAIL. Overexpression of MARCH2 exhibits tumor suppressive properties and phenocopies the effects of SNAIL downregulation and PTK6 inhibition in TNBC cells, such as inhibition of migration, anoikis resistance, and metastasis. Consistent with this, higher levels of MARCH2 expression in breast and other cancers are associated with better prognosis. We have identified MARCH2 as a novel SNAIL E3 ligase that regulates EMT and metastases of mesenchymal TNBC.

**Significance:**

EMT is a process directly linked to drug resistance and metastasis of cancer cells. We identified MARCH2 as a novel regulator of SNAIL, a key EMT driver, that promotes SNAIL ubiquitination and degradation in TNBC cells. MARCH2 is oncogene regulated and inhibits growth and metastasis of TNBC. These insights could contribute to novel strategies to therapeutically target TNBC.

## Introduction

Treatment resistance, recurrence, and metastases are responsible for the majority of breast cancer–related deaths. Epithelial-to-mesenchymal transition (EMT) in cancer is associated with chemotherapy resistance and enhanced dissemination (1, 2). As a result of EMT, epithelial-like cancer cells lose cell-cell contacts, gain migratory and invasive capacity, and acquire anoikis resistance, thereby enhancing their metastatic capacity. Cancer cells that acquire mesenchymal phenotype exhibit cancer stem cell–like properties and are also relatively more resistant to chemotherapy and radiotherapy (3, 4). Furthermore, mesenchymal cancer cells exhibit changes in expression of immunomodulatory molecules that could impact the interaction of tumor cells with their immune microenvironment and modulate response to immunotherapies (5, 6).

EMT can be triggered by several distinct mechanisms, including cell stress, extracellular stimulation, signaling pathways, miRNA regulation, and transcriptional regulation (2, 6). Among EMT transcriptional regulators, members of the Snail family potently drive an EMT program in cancer cells. As a transcriptional corepressor of E-cadherin, SNAIL directly suppresses *CDH1*, a gene encoding E-cadherin, via its zinc finger domain by binding to E-box elements of the CDH1 promoter (7). Suppression of E-cadherin expression in cancer cells is a key characteristic of EMT leading to the mesenchymal phenotype (8, 9). Furthermore, SNAIL in breast cancer cells may promote expansion of a stem cell–like population that is resistant to apoptosis and exhibit self-renewal capacity (10). SNAIL expression is also prognostic in multiple tumor types, with higher levels associated with recurrence, metastasis, and resistance to chemotherapy and radiotherapy (11, 12).

SNAIL is regulated transcriptionally, as well as by posttranslational modifications, such as acetylation, phosphorylation, and ubiquitination (13–15). Ubiquitin proteasome–dependent SNAIL degradation is promoted by several established mechanisms: (i) phosphorylation of SNAIL in the serine-rich domain (SRD) by GSK3β leads to β-TRCP/FBW1-dependent ubiquitination (16); (ii) PKD1-dependent phosphorylation of SNAIL in the Snag domain (Ser11) enhances FBXO11-dependent SNAIL ubiquitination (17); and (iii) GSK3β phosphorylation–independent degradation promoted by FBXL5 and FBXL14 E3 ligases (18, 19). In contrast, acetylation of SNAIL prevents its recognition by E3 ubiquitin ligases such as FBXL14 and β-TRCP, thereby preventing SNAIL polyubiquitination and degradation (20).

We previously identified SNAIL as a critical mediator of EMT and metastasis of triple-negative breast cancer (TNBC) cells driven by the non-receptor protein tyrosine kinase (PTK6; ref. 21). PTK6 is highly expressed in about 45% of TNBC patient tumors and activated by autophosphorylation at tyrosine 342 (21, 22). Overexpression of PTK6 is sufficient to drive a partial EMT by stabilizing SNAIL in a kinase activity–dependent manner (21). PTK6 downregulation or kinase inhibition promotes SNAIL degradation and EMT reversal via a novel mechanism independent of known SNAIL E3 ligases, sensitizing TNBC cells to anoikis cell death and suppressing their metastasis. In the current study, we used a screening approach to identify MARCH2 (membrane-associated RING-CH2) as a novel PTK6-dependent, SNAIL E3 ligase that promotes SNAIL ubiquitination and degradation. In addition, MARCH2 overexpression suppresses growth, invasion, and metastasis of TNBC, exhibiting tumor suppressive properties, and phenocopying effects of PTK6 or SNAIL inhibition. Our findings provide mechanistic insight into how PTK6 regulates proteosome-dependent degradation of SNAIL and further support PTK6 as a novel targetable EMT regulator.

## Materials and Methods

### Antibodies and Reagents

Antibodies were purchased from Cell Signaling Technology: HA (RRID:AB_1549585, 10691311), Flag (RRID:AB_2572291) SNAIL (RRID:AB_2255011, 2191759), GAPDH (RRID:AB_10622025), Zeb1 (RRID:AB_2935802), Slug (RRID:AB_2239535), Vimentin (RRID:AB_10695149), PTK6 (RRID:AB_2799479), β-TRCP (RRID:AB_10545763), and H3 (RRID:AB_10544537). MARCH2 antibody (RRID:AB_2548094) was purchased from Thermo Fisher Scientific. For IHC, the following antibodies were purchased from Sigma-Aldrich: anti-PTK6 (BRK) rabbit IgG (RRID:AB_10669982), anti-SNAIL rabbit IgG, (SAB5700796). PTK6 kinase inhibitors P21d and 4f were purchased from Tocris and Millipore Sigma (531000), respectively, and TWS119 was purchased from Santa Cruz Biotechnology.

### siRNA/short hairpin RNA

siRNA for PTK6 (catalog no. D-003166-06) and MARCH2 (SMART Pool and individual sequences, catalog no. M-006986-02, D-006986-02, D-006986-18) were purchased from Dharmacon and transfection was performed using Oligofectamine (Thermo Fisher Scientific), following the manufacturer's protocol and as described previously (21).

Human PTK6 (NM_005975) short hairpin RNAs (shRNA) were purchased from Sigma-Aldrich and are identified in the figures by the last two digits of the TRC numbers: TRCN0000196912 [12] and TRCN0000021552 [52]. Human MARCH2 shRNA viral particles were purchased from Origene (TL315142V) with the following targeting sequences: (i) CTGTCTGGAGAAGTGGC TTTCCTCATCTA; (ii) GCTACTGCGAGCTGTGCCACACGGAGTTT.

### Cell Culture, Transfection, Virus Generation, and Infection

MDA-MB-231 cells were obtained from ATCC (RRID:CVCL_0062) and cultured in RPMI 1640 media supplemented with 10% FBS, HEPES buffer, and Pen/Strep antibiotics. MDA-MB-231/LM2-4 cell lines were obtained from Robert Kerbel (Sunnybrook Institute, Ontario) and cultured in RPMI 1640 media supplemented with 5% FBS and HEPES buffer. MMTV-myc cells were obtained from Eduardo Farias (Mount Sinai) and cultured in DMEM F12 media supplemented with 5% FBS, insulin, and 1% Pen/Strep. All cell lines were routinely tested for *Mycoplasma* using MycoStrip (Invivogen) and were confirmed negative as of December 2023 using MycoStrip (Invivogen). Cells lines were authenticated using ATCC's short tandem repeat Profiling Service and confirmed to be triple negative by Western blot analysis. All cells were used within 10 passages after thawing.

### Plasmids, Transfection, and Virus Production

Plasmids or viral supernatants were obtained from the following: pPGS-SNAIL-Flag (RRID:Addgene_25695); pLEX-MSC-SNAIL Firefly-luciferase (F-Luc) and pMSCV-hygro-Renilla-Luc (Yibin Kang, Princeton University, Princeton, NJ); pLv117-Control-3HA (GeneCopoeia EX-NEG-Lv117); pLv117-MARCH2-3HA (GeneCopoeia EX-K4473-Lv117); pcDNA3-HA-ubiquitin (RRID:Addgene_18712); pCMV-ubiquitin-3HA (Zhen-Qiang Pan, Mount Sinai); pLenti-puro-3HA-ubiquitin (RRID:Addgene_742198); pEB-GST-MARCH2-WT, pEB-GST-MARCH2-A97 and pEB-GST-MARCH2-tm (Ramnik Xavier, Massachusetts General Hospital); pCMV-MARCH2 (Origene SC114251); pCMV-BTRCP (Origene SC110015); pFUO-mCherry-luc (Adolfo Ferrando, Columbia University, New York, NY); pLenti-C-mGFP-MARCHF2 (Origene RC207517L4V).

To generate mutant MARCH2 lentiviral vectors, QuikChange II XL Site-Directed mutagenesis kit (Agilent Technologies) was used according to the manufacturer's protocol. Primers for mutagenesis were: (Forward) GGT GTT AGA TGA GGA AAG CGC CTT CTC CAG ACA GCT CTT and (Reverse) AAG AGC TGT CTG GAG AAG GCG CTT TCC TCA TCT AAC ACC. All mutagenesis was sequence confirmed (Genewiz).

Transfection of plasmids was performed using Lipofectamine 2000 (Thermo Fisher Scientific), following the manufacturer's protocol. To generate lentivirus, HEK293T cells (ATCC, catalog no. CRL-3216) were cotransfected with lentiviral vector and vectors encoding VSV-G envelope and delta8.9, as described previously (21). Retrovirus was generated using GPG-293T cells, as described previously (21, 23). Culture supernatant containing virus was collected daily, filtered and applied to target cells, along with 2 µg/mL hexadimethrine bromide (polybrene) and cells were subsequently placed in antibiotic selection medium.

### Ubiquitin Assay, Co-immunoprecipitation, and Western Blot Analysis

MDA-MB-231 or HEK293T cells overexpressing FLAG-SNAIL were transfected with MARCH2 or β-TRCP as well as 3HA-tagged ubiquitin plasmids. After 48 hours, cells were treated with 20 µmol/L MG132 (Sigma-Aldrich) for 4 hours and then lysed in denaturing lysis buffer (1.5% SDS, N-ethylmaleimide and phenylmethylsulfonylfluoride in TBS, pH7.4). Lysates were incubated at 95°C for 15 minutes, followed by immunoprecipitation procedure. For co-immunoprecipitation, FLAG-SNAIL–expressing MDA-MB-231 cells were lysed in NP40 lysis buffer (Boston Bioscience). Cell lysates (0.5 µg total protein in 1 ml of lysis buffer) were precleared with mouse IgG-conjugated agarose (Sigma-Aldrich), followed by incubation with anti-flag EZView Red M2 affinity gels (catalog no.: F2426, Sigma-Aldrich). After 1-hour incubation at room temperature, the beads were washed three times and incubated at 95°C for 10 minutes in 2x sample buffer. Eluates were resolved on NuPAGE 4%–12% Bis-Tris protein gels (Thermo Fisher Scientific) and transferred onto polyvinylidene difluoride membrane. Western blot analysis was performed with primary antibody according to manufacturer's protocol. Quantification of band intensity was performed using ImageJ software.

The *in vitro* ubiquitination reactions were performed using 2 µmol/L recombinant human SNAIL protein (Origene TP304581), 3 µmol/L recombinant human March2 E3 ligase (Origene TP307517), 100 µmol/L Ubiquitin (R&D Systems U-100H-10M), 100 nmol/L recombinant E1(UBE1, Biotechne E-305), 500 nmol/L recombinant E2 (UBE2D3, Biotechne E2-627), 1X ATP Energy Regeneration buffer (Enzo BML-EW9810-0100) in a 25 µL reaction buffer containing 50 mmol/L Hepes, pH 8.0, 50 mmol/L NaCl, and 1 mmol/L TCEP. After 1 hour at 37°C, reactions were stopped with 4X Laemmli SDS-Sample Buffer and boiled at 95°C for 5 minutes.

### Proximity Ligase assay with Duolink

Proximity ligase assay (PLA) was performed using Duolink In Situ Kit (Sigma-Aldrich) according to the manufacturer's protocol. MDA-MB-231 cells cultured on coverslips in 12-well plates were fixed in 4% paraformaldehyde/PBS for 20 minutes and blocked/permeabilized with 5% BSA/0.1% Triton X in PBS for 1 hour at room temperature. Cells were incubated with primary antibody overnight at 4°C, washed and incubated with PLA probe anti-rabbit PLUS and anti-mouse MINUS Donkey IgG (RRID: AB_2713942) at 37°C for 1 hour. After wash, ligase reaction was performed followed by amplification with Red detection reagent. AlexaFluor 488 Phalloidin (Thermo Fisher Scientific) was used for counter staining actin and mounted with VECTASHIELD with DAPI (Vector Laboratories). Images were captured by a SP5 Leica confocal microscope with a fixed setting for PLA signal (red). The number of PLA signals per cells (*n* = 90 to 300 cells) was calculated using ImageJ software.

### IHC

Tumor tissue microarrays were obtained from Icahn School of Medicine at Mount Sinai Department of Pathology. Mouse tumor tissues were fixed in 4% paraformaldehyde and embedded in paraffin (FFPE) or directly frozen in optimal cutting temperature compound. FFPE slides were deparaffinized in xylene substitute (Sigma-Aldrich) and hydrated in ethanol/H_2_O solution (100%, 90%, 75%, 50% ethanol/H_2_O and 100% H_2_O). Slides were incubated in citrate-based antigen retrieval buffer (Sigma-Aldrich) in a steamer and blocked with BLOXALL (Vector Laboratories) for 15 minutes at room temperature. Slides were incubated with primary antibody, washed, then incubated with ImmPRESS horseradish peroxidase–conjugated secondary antibody (Vector Laboratories), followed by ImmPACT 3,3′-Diaminobenzidine (DAB) peroxidase substrate (Vector Laboratories). Peptide competition assays were performed using PrEST antigens (PTK6: catalog no. APREST78020, Sigma-Aldrich). The slide images were captured by Panoramic 250 FLASH II digital scanner (Perkin Elmer) or Leica DM550 fluorescence microscope and analyzed by CaseViewer (3DHISTECH Ltd.) or LAS AF software. The staining intensity was scored by pathologists as low (negative or +) or high (++ or +++).

### siRNA Screening

MDA-MB-231 cells expressing both pLEX-SNAIL-F-luc and pMSCV-R-luc (2.5 × 10^4^ cells per well) were seeded into 96-well plates. siRNA transfection was performed using Oligofectamine and siRNA library (ubiquitin conjugate sets 1–3, Dharmacon, catalog nos. G-005615-01, G-005625-01, G-005635-01) or siRNA controls (OTC non-targeting control and siGENOME non-targeting control #5, Dharmacon). Media was replaced with DMSO or P21d-containing media. After 24 hours, the dual luciferase assay (DLA) was performed with Dual-Glo Luciferase Reagent and GloMAX Discover System (Promega). The F-luc/R-luc (*Renilla*-luciferase) values of P21d-treated wells were normalized to DMSO-treated wells. SNAIL recovery rate by siRNA gene X was calculated by follow (control in each plate). Recovery rate (fold change) = [Snail reduction % by siRNA X)]/[Snail reduction % by siRNA Control #5). For secondary screening, Western blot analysis was performed using antibodies against FLAG (RRID:AB_2572291) and GAPDH. Tertiary screening was performed using DLA of parental MDA-MB-231 cell transfected with top candidate siRNA.

### Transwell Migration Assay and Boyden Chamber Invasion Assay

Transwell migration assay was performed as described previously (21). Migrated MDA-MB-231 cells were stained with Protocol HEMA3 (Thermo Fisher Scientific), and cell number was counted by ImageJ software. Invasion assay was performed with 96-well CytoSelect 96-well Cell Invasion assay Basement Membrane kit (Cell BioLabs)

### Anoikis Assay

MDA-MB-231 cells were incubated in suspension condition for 24 or 48 hours at 5% CO_2_ at 37°C. The cells were washed in PBS and stained with FITC-AnnexinV/PI (propidium iodide) staining kit (BD Biosciences). The cells were then analyzed by flow cytometer (BD FACSCanto or LSRFortessa) and De Novo FCS Express software.

### Three-dimensional Matrigel Culture Assay

For three-dimensional (3D) culture assay, 96-well plates were coated with 55 µL of growth factor reduced Matrigel Matrix (Corning). A total of 3,000 cells were seeded on top of the solidified Matrigel. After 5 days, the cells were visualized, and the cell viability was measured by CellTiter-Glo 3D Viability Assay (Promega).

### Quantitative RT-PCR

Total RNA was isolated using RNEasy Mini Kit (Qiagen). Synthesis of cDNA was carried out using Superscript IV (Life Technologies) using the manufacturer's instructions. Total RNA (1 µg) was reverse transcribed with SuperScript IV (Invitrogen) using random hexamers (Promega) according to the manufacturer's protocol. The resulting cDNA was diluted 1/1,000 and 1 µL cDNA was used as a PCR template. Primers used to amplify *March2*, and the loading control β-*actin* are listed below. qPCR reactions were assembled using Power SYBR Green PCR Master Mix (Life Technologies) according to the manufacturer's protocols and quantified using Quant Studio 7 Pro (Applied Biosystems) qPCR machine. Expression fold changes were calculated using the ∆∆Ct method. Primers used were: *MARCH2* (forward) CAGAGGAGACACCAGTATGAATG, *MARCH2* (reverse) GTTGAAGTGGAAGTGTTGAAGTG, β-*actin* (forward) CACCATTGGCAATGAGCGGTTC, β-*actin* (reverse) AGGTCTTTGCGGATGTCCACGT.

### Patient Samples

Samples from patients treated at the Mount Sinai Hospital were collected for patient-derived xenograft (PDX) generation and expansion. Written informed consent was obtained for all subjects on Icahn School of Medicine at Mount Sinai (ISMMS) Institutional Review Board (IRB)-approved protocol (HSM# 14-00330) prior to the procedure at which the specimen was obtained. The studies were conducted in accordance with the Belmont Report and U.S. Common Rule and approved by the ISMMS IRB.

### Metastatic Lung Colonization and Spontaneous Metastases Assays

All animal procedures were conducted in compliance with the guidelines of the Institutional Animal Care and Use Committee of Icahn School of Medicine at Mount Sinai under approved protocol LA12-00113. In our experimental metastasis models, 8–10 weeks old female NSG mice (Jackson Laboratory, RRID: IMSR_JAX:005557) were randomized and injected intravenously (tail vein) with 1 × 10^6^ cells/100 µL of either MDA-MB-231/Luciferase cells overexpressing control vector (*N* = 8), wild-type (WT) MARCH2 (*N* = 9) or RING domain mutant (W97A) MARCH2 (*N* = 12). Lung metastases were imaged using *in vivo* imaging system (IVIS) 7 and 14 days after injection (Translational and Molecular Imaging Institute at Mount Sinai). During IVIS imaging, mice were anesthetized with inflowing isoflurane (2%–2.5%) in 2 to 2.5 L/minute oxygen. After anesthesia, 10 µL/g mouse body weight of Luciferin solution (Revvity Inc.) was intraperitoneally injected into mouse. IVIS images taken 15 minutes after Luciferin injection were used for image analysis. The IVIS images were analyzed using Living image software (Version 4.7.3, Revvity Inc. RRID: SCR_014247). A 3.3 × 3.3 cm^2^ area in the lungs was set as region of interest (ROI) for each mouse, and then total photons/second in the ROI was obtained. Statistical difference between the groups was assessed by Student *t* test, and the *P* value less than 0.05 was regarded as statistically significant.

For the spontaneous metastasis models, 8–10 weeks old female NSG mice were randomized to be injected with 5 × 10^5^ MDA-MB-231/LM2-4 Luciferase cells overexpressing control vector (*N* = 10) or WT MARCH2 (*N* = 10) into the left fourth mammary fat pad. Primary tumor growth was monitored by caliper measurements every day beginning the seventh day after implantation and tumor volume was calculated using the formula: ½ (width^2^  ×  length). Lung metastases were imaged 12, 15, and 18 days after injection using IVIS and analyzed as above. During IVIS imaging, the primary tumor site of the mice was shielded with opaque black paper.

### Data Analysis and Statistical Analysis

All values were expressed as mean ± SEM unless indicated otherwise, and the number of samples (*n*) was shown. Experiments were repeated minimum of three, unless indicated otherwise. The statistical significance of differences between groups was determined by Student *t* test with *P* < 0.05 considered significant (*, *P* < 0.05; **, *P* < 0.01; ***, *P* < 0.001). F-test was also performed to confirm similar variances of groups. Two-way ANOVA with the Bonferroni *post hoc* test was performed on the tumor growth experiments. All statistical analyses were performed using GraphPad Prism v6.0.

### Survival Curves

#### The Cancer Genome Atlas Data

RNA sequencing (RNA-seq) of breast cancer data from The Cancer Genome Atlas (TCGA; RRID:SCR_003193; ref. 24) were downloaded from Genomic Data Commons (GDC) data portal (https://portal.gdc.cancer.gov/). We downloaded mapped and gene level–summarized (level 3, reads per kilobase of transcript per million mapped reads) RNA-seq profiles. log_2_ transformation was performed after adding a count of 1 to each value. The log_2_ transformed values were used for further analysis.

#### Molecular Taxonomy of Breast Cancer International Consortium Data

The Molecular Taxonomy of Breast Cancer International Consortium (METABRIC) data breast cancer dataset (PMID: 22522925) was downloaded through the European Genome–Phenome Archive (https://ega-archive.org/, study ID EGAS00000000083) and consists of 1,302 breast tumors with matching detailed clinical annotations, long-term follow-up, expression data. The mRNA expression was profiled using Illumina HT-12 v3 platforms. The normalized mRNA expression data were downloaded.

#### Association with Clinical Outcomes

We tested for the overall survival (OS) associations with MARCH2 expression levels. We used Cox proportional hazards regression with MARCH2 expression levels. We also separated samples based on whether their MARCH2 expression level was higher than upper quartile or lower than lower quartile. We fit Kaplan–Meier (KM) survival curves and tested the equivalence of the curves using log-rank tests for high versus low expression levels.

We used the same method for testing OS association with SNAI1, SNAI2, TWIST, and ZEB1 expression levels in patients with TNBC. For these associations, we separated samples as high versus low expressed samples based on median expression levels.

#### Meta-analysis Using KM Plotter

The relationship between SNAI1 expression levels and prognosis of patients with TNBC was additionally analyzed using KM plotter (RRID:SCR_018753). The KM plotter database contains survival data of patients with TNBC. Patients were split according to median expression level of SNAI1 expression levels, and recurrence-free survival of the two groups was compared and tested using log-rank tests.

### Data Availability

The data analyzed in this study were obtained from GDC data portal (https://portal.gdc.cancer.gov/), the European Genome–Phenome Archive (https://ega-archive.org/, study ID EGAS00000000083), and KM plotter (RRID:SCR_018753).

## Results

### SNAIL Expression has Prognostic Significance within TNBC Subtype and Correlates with PTK6 Oncogene Expression

**FIGURE 1:**
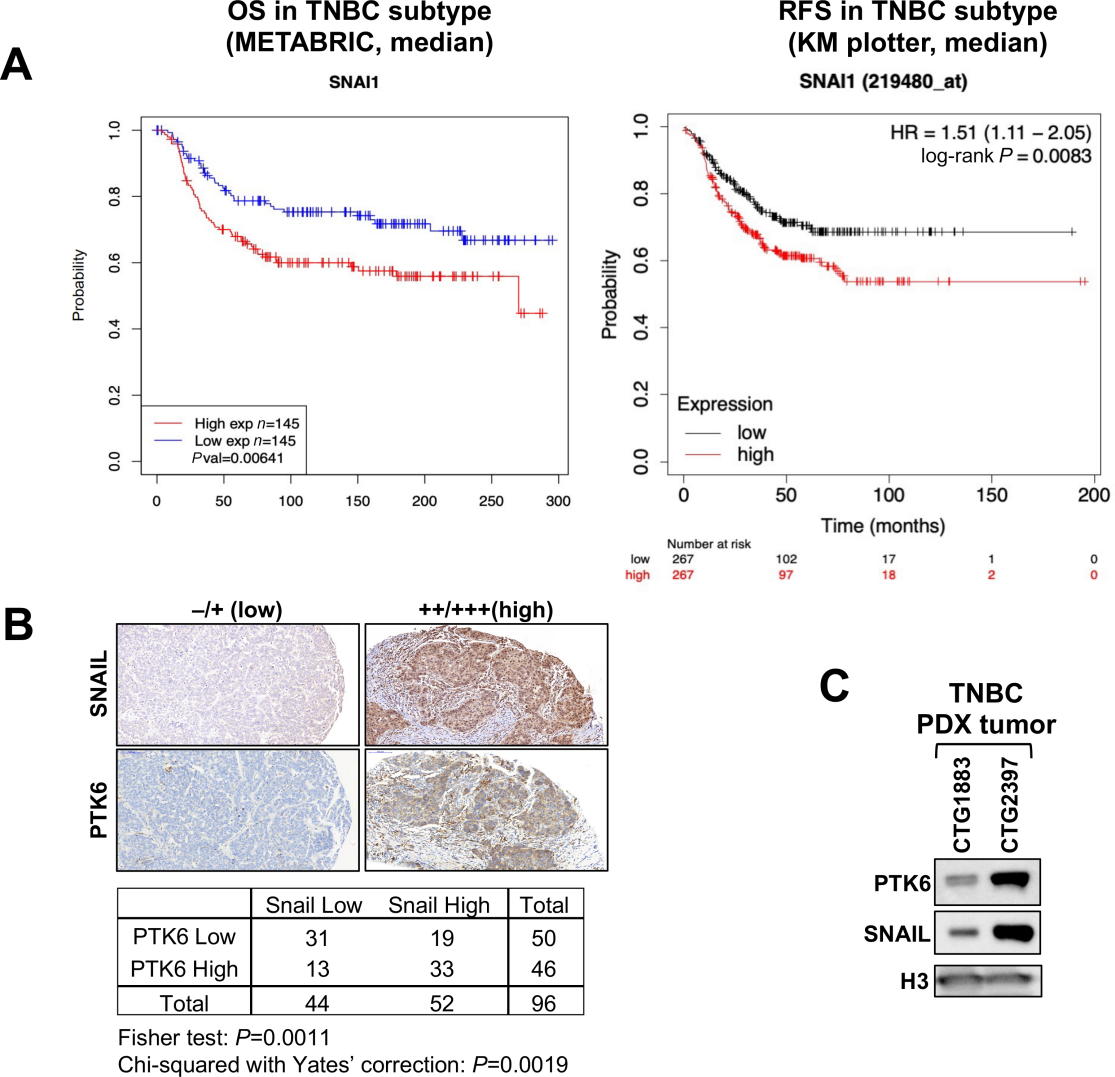
Expression of SNAIL and PTK6 in TNBC. **A,** Prognostic significance of SNAIL expression in TNBC. KM survival curves of SNAIL expression in patients with TNBC (defined as estrogen receptor, progesterone receptor, and HER2 negative) using the METBRIC dataset (*n* = 290) and KM plotter (*n* = 734). SNAIL expression was divided along the median into low versus high expressors. **B,** Representative images of IHC staining for PTK6 and SNAIL expression in TNBC tissue microarray (*n* = 48). Low (0, 1+) and high (2+, 3+) expression was scored by two independent reviewers. **C,** Expression of PTK6 and SNAIL in triple-negative PDX tumors.

As a transcriptional corepressor of E-cadherin, SNAIL is sufficient to promote EMT. EMT is associated with increased invasiveness, chemotherapy resistance, and metastatic potential (1, 2, 4). Levels of SNAIL in TNBC have prognostic significance. In the METABRIC dataset of 290 TNBCs, a higher level of *SNAIL* expression is associated with worse OS (log-rank *P* = 0.00641; Fig. 1A).

Similarly in a meta-analysis of patients diagnosed with TNBC (PMID: 34309564), a higher *SNAI1* transcript level is associated with worse relapse-free survival (with low *n* = 267/high *n* = 267, log-rank *P*-value = 0.0083; Fig. 1A). Interestingly, this prognostic significance is specific for SNAIL among EMT driver genes, as levels of *SLUG*, *ZEB1* or *TWIST* are not associated with prognostic significance (Supplementary Fig. S1).

We previously identified PTK6 as a novel oncogenic regulator of SNAIL that stabilizes SNAIL protein expression in TNBC (21). PTK6 and SNAIL protein levels are correlated in patient with TNBC, as assessed by IHC analysis of patient with TNBC. In a tissue microarray of 48 TNBC (double cores) patient tissues, a high level of SNAIL expression (2+ or 3+) was detected in about 50% of cases and was positively correlated with high staining scores for PTK6 (Chi-squared with Yate's correction = 0.0019; Fig. 1B). Furthermore, TNBC PDX tumors with higher levels of total PTK6 also express higher levels of SNAIL protein (Fig. 1C). These results further support our previous finding that PTK6 enhances SNAIL expression in TNBC breast cancer cells.

### PTK6 Inhibition Promotes Ubiquitination and Degradation of SNAIL

**FIGURE 2:**
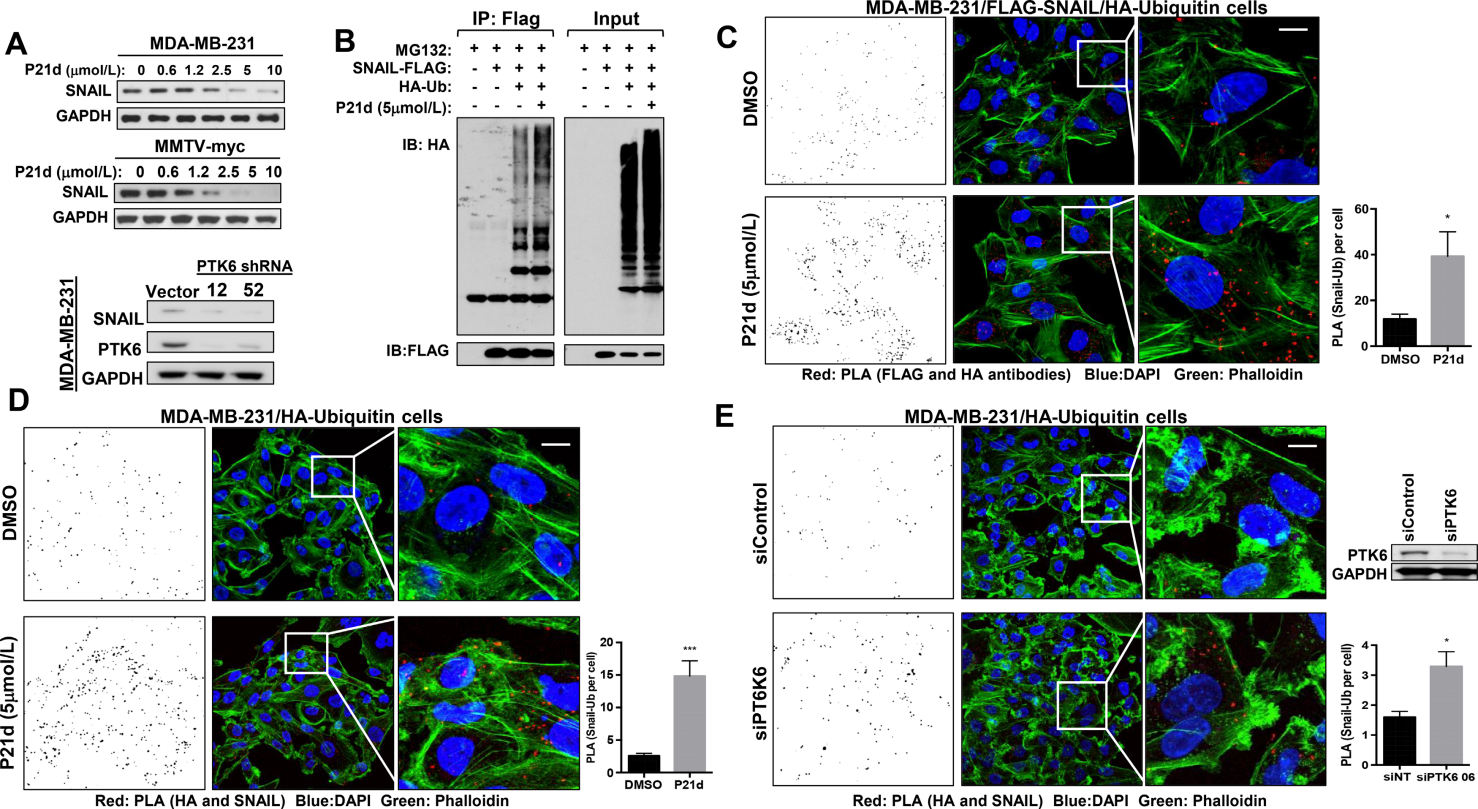
PTK6 inhibition increases ubiquitination and degradation of SNAIL in TNBC. **A,** PTK6 inhibitor (P21d) treatment or PTK6 shRNA downregulates SNAIL in TNBC cells. Human MDA-MB-231 or mouse MMTV-myc cells were treated with P21d for 24 hours and SNAIL expression was assessed by Western blot analysis. Lysates of MDA-MB-231 cells expressing vector control or either of two independent PTK6 shRNA vectors were assessed for SNAIL expression. **B,** MDA-MB-231 cells overexpressing HA-ubiquitin and Flag-SNAIL were treated with P21d for 8 hours with MG132 cotreatment for the last 4 hours. Lysates were immunoprecipitated with anti-FLAG antibody and probed with anti-HA or FLAG antibody. The result is representative of three independent experiments. **C,** Representative images of Duolink PLA using MDA-MB-231 cells coexpressing HA-ubiquitin and Flag-SNAIL. Cells were treated with P21d (5 µmol/L) for 24 hours with MG132 cotreatment for the last 4 hours and incubated with antibodies against HA (Ubiquitin) and FLAG (SNAIL) for PLA (red) and Phalloidin for F-actin and counterstaining. PLA signals (red)/cell were quantitated using ImageJ (left). Scale bar, 15 µm. **D,** Representative images of PLA using MDA-MB-231 cells overexpressing HA-ubiquitin only. Cells were treated with P21d for 24 hours and with MG132 cotreatment for the last 4 hours and incubated with antibodies against HA (Ubiquitin) and SNAIL for PLA (red) and Phalloidin for F-actin counterstaining. PLA signals/cell were quantitated using ImageJ (left). Scale bar, 15 µm. **E,** Representative images of PLA using MDA-MB-231 cells overexpressing HA-ubiquitin only that were transfected with control or PTK6 siRNA for 48 hours. Cells were treated with MG132 for the last 4 hours and incubated with antibodies against HA (Ubiquitin) and SNAIL for PLA (red) and Phalloidin for F-actin counterstaining. PLA signals/cell were quantitated using Image (left). Scale bar, 15 µm. All the PLA signals were counted by ImageJ with a fixed setting. Data represent mean ± SEM. *, *P* = 0.05; ***, *P* = 0.0001 by Student *t* test.

PTK6 is activated and autophosphorylated in patient with breast cancers and breast cancer cell lines (21, 25). Downregulation of PTK6 or inhibition of its kinase activity promotes degradation of SNAIL in a proteasome-dependent manner and partially reverses EMT of TNBC cells (21). Treatment with PTK6 kinase inhibitor (P21d or 4f) or PTK6 shRNA downregulates SNAIL expression in human and mouse TNBC cells (Fig. 2A; Supplementary Fig. S2; ref. 21). Treatment with P21d promotes ubiquitination of SNAIL in triple-negative MDA-MB-231 cells, as assessed biochemically and by immunofluorescence using Duolink PLAs (Fig. 2B–D). With PLA, a signal is detected only when there is close interaction or colocalization of molecules. PLA can also be adapted to detect posttranslational modifications on individual molecules such as ubiquitination and SUMOylation (26, 27).

P21d treatment increases ubiquitination of FLAG-tagged SNAIL immunoprecipitated from cells MDA-MB-231 cells coexpressing HA-Ubiquitin (Fig. 2B). In addition to these biochemical assessments, PLA was used to quantitate the levels of SNAIL ubiquitination by immunofluorescence. We validated PLA as a methodology to detect SNAIL ubiquitination in cells overexpressing an established SNAIL E3 ligase, β-TRCP, using antibodies for SNAIL and Ubiquitin (16). β-TRCP overexpression increases ubiquitination of immunoprecipitated FLAG-SNAIL (Supplementary Fig. S3A). In β-TRCP–overexpressing cells, increased ubiquitin/SNAIL PLA signals were detected using two independent mixes of anti-HA (for ubiquitin) and anti-SNAIL antibodies (Supplementary Fig. S3B and S3C). Furthermore, these PLA signals were suppressed by GSK3β inhibitor (TWS119) treatment, as GSK3β phosphorylation is a requisite step for β-TRCP–dependent ubiquitination (Supplementary Fig. S3D).

PTK6 kinase activity inhibition or PTK6 downregulation increased SNAIL ubiquitination, as assessed by PLA. PTK6 kinase inhibitor P21d treatment increased SNAIL:Ubiquitin signals in cells overexpressing FLAG-SNAIL and HA-Ubiquitin by 2.3-fold (Fig. 2C). A 6.0-fold increase in endogenous SNAIL:Ubiquitin PLA signals was detected in MDA-MB-231 cells expressing only HA-Ubiquitin treated with P21d compared with vehicle control (Fig. 2D). Finally, increased endogenous SNAIL:Ubiquitin PLA signals were detected in MDA-MB-231 cells expressing HA-Ubiquitin transfected with PTK6 siRNA compared with control siRNA (Fig. 2E). These data collectively support an increase in SNAIL ubiquitination following PTK6 inhibition or downregulation.

### Novel PTK6-dependent Regulators of SNAIL Ubiquitination and Degradation

**FIGURE 3:**
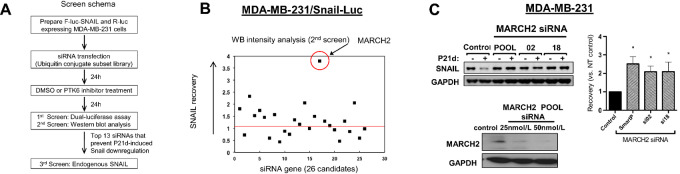
MARCH2 is a novel PTK6-regulated SNAIL E3 ligase. **A,** Schema of siRNA screening strategy used to identify PTK6-regulated SNAIL E3 ligases. **B,** MARCH2 siRNA (SMART Pool) identified as top candidate siRNA that restores SNAIL expression in P21d-treated MDA-MB-231/SNAIL- Luciferase cells by Western analysis. **C,** MARCH2 siRNA (SMART POOL or individual) prevents P21d-induced SNAIL downregulation in MDA-MB-231 cells.

SNAIL ubiquitination and proteasome-dependent degradation downstream of PTK6 inhibition is independent of known mechanisms. We previously reported that established SNAIL E3 ligases such as GSK3β/β-TRCP, FBXL5, FBXL14, and FBXO11 have no role in PTK6-dependent regulation of SNAIL; downregulation of these E3 ligases did not impact SNAIL levels in P21d-treated TNBC cells in our previous report (21). To identify the novel E3 ligase(s) responsible for PTK6-dependent SNAIL regulation, an siRNA library of 588 E3 ligases and related proteins was screened to identify those siRNAs that restored Snail levels in P21d-treated TNBC cells. This strategy would identify molecules that are critical for P21d-induced, but not basal, SNAIL ubiquitination and degradation (Fig. 3A).

To enable primary screening using luciferase assays, MDA-MB-231 cells were engineered to express SNAIL protein fused to F-luc in addition to R-luc, which serves as control for changes in viability (gift of Yibin Kang, Princeton). P21d treatment of these cells was confirmed to decrease levels of exogenously expressed SNAIL-F-luc as assessed by Western blot analysis and Dual-glo luciferase assay (Supplementary Fig. S4A). SNAIL-F-luc/R-luc–expressing MDA-MB-231 cells were transfected with the libraries of SMART Pool siRNAs. Beginning 24 hours after transfection, cells were treated with P21d or DMSO vehicle control for 24 hours and the dual Luciferase reporter assay (Promega) was used to assess levels of SNAIL-F-Luc and R-Luc. From the primary screen, 26-candidate siRNAs were identified that prevented downregulation of SNAIL-F-Luc in P21d-treated cells but did not affect basal SNAIL-F-Luc levels in DMSO-treated cells. The effect of these 26-candidate siRNAs on SNAIL-F-luc protein expression in P21d-treated cells was confirmed by Western blot analysis (secondary screening; Fig. 3B). Quantification of the immunoblot intensity shows that 13-candidate siRNAs led to recovery of SNAIL-F-Luc levels in P21d-treated cells (Fig. 3B; Supplementary Fig. S4B).

The 13 prioritized SMART Pool siRNAs were assessed for effects on endogenous SNAIL levels in P21d- or DMSO-treated MDA-MB-231 cells (tertiary screen) and MARCH2 emerged as the top PTK6-regulated SNAIL E3 ligase candidate (Fig. 3C). Transfection with either MARCH2 SMART Pool siRNA or either of two individual MARCH2 siRNA (02 and 18) restores SNAIL expression in P21d-treated cells. These results support MARCH2 as a novel PTK6-regulated E3 ligase candidate that promotes proteasome-dependent SNAIL degradation.

### MARCH2 is a Novel SNAIL E3 Ligase Regulated by PTK6 Kinase Activity

MARCH2 is a member of the RING family of E3 ligases with an increasing number of target substrates. The MARCH family of proteins, first discovered as proteins encoded by Kaposi sarcoma–associated herpes viruses, now consists of 11 members that contain a variable number of putative transmembrane domains and an N-terminal cytoplasmic RING-CH domain with E3 ubiquitin ligase activity (28–33). MARCH2 substrates identified thus far include β2 adrenergic receptor, cystic fibrosis transmembrane regulator (CFTR), DLG1, syntaxin-6, and Disheveled (34–38).

**FIGURE 4:**
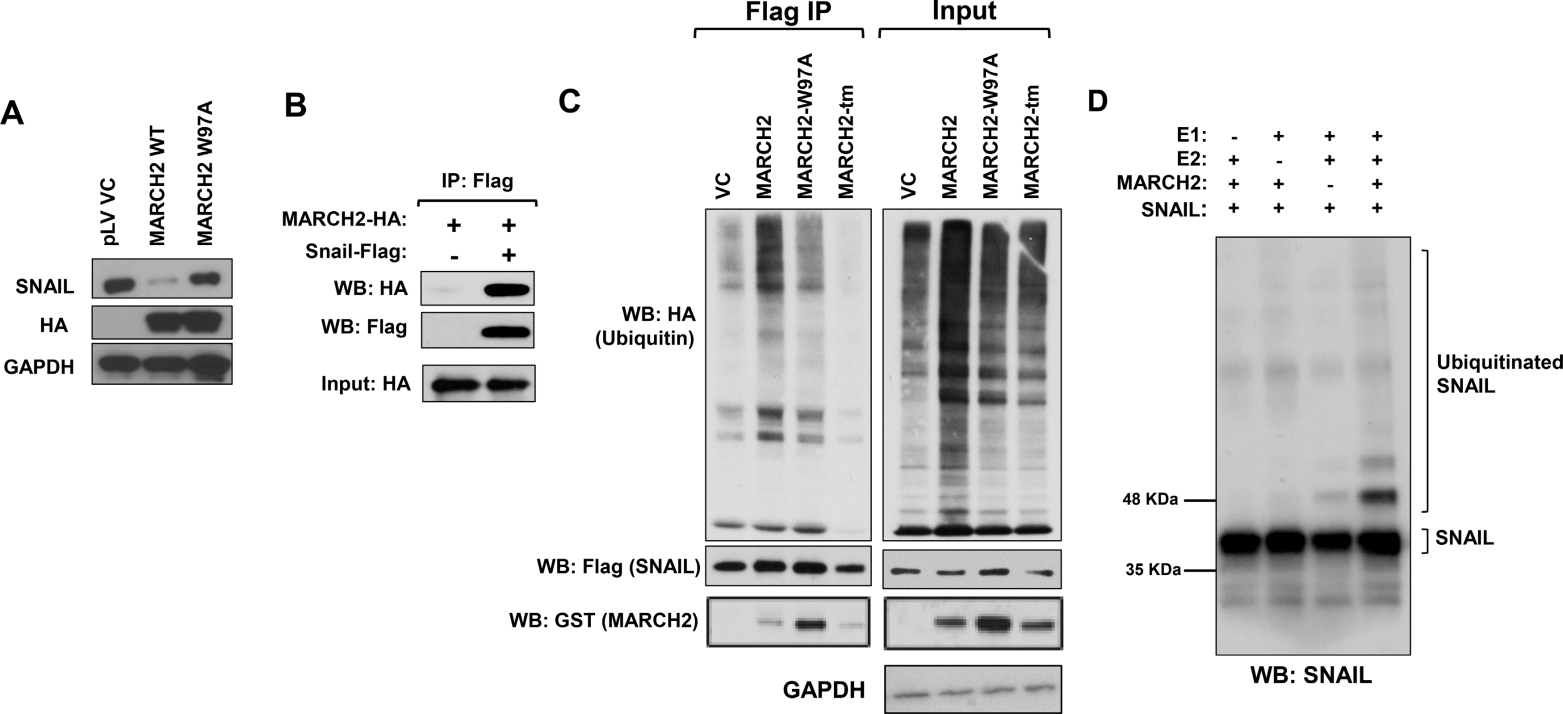
MARCH2 promotes ubiquitination and downregulation of SNAIL. **A,** HA-tagged WT, but not RING domain–mutant (W97A), MARCH2 downregulates SNAIL levels in MDA-MB-231 cells. **B,** MARCH2 coprecipitates with SNAIL in MDA-MB-231 cells coexpressing FLAG-SNAIL and HA-MARCH2. **C,** WT, but not RING domain– or transmembrane domain–mutant, MARCH2 ubiquitinates SNAIL. FLAG-Snail was immunoprecipitated from MDA-MB-231 cells coexpressing FLAG-Snail, HA-Ubiquitin and GST-tagged MARCH2 constructs. Immunoprecipitates were blotted with antibodies to HA to assess ubiquitination. **D,** MARCH2 ubiquitinates SNAIL in *in vitro* ubiquitination assay. Reactions were performed with recombinant SNAIL (Myc-tagged) in the presence of ubiquitin, recombinant E1 (UBE1), recombinant E2 (UBE2D3) and/or recombinant MARCH2.

Overexpression of WT MARCH2, but not RING domain–mutated MARCH2 (W97A), downregulates endogenous SNAIL expression in MDA-MB-231 cells (Fig. 4A). MARCH2 expression did not affect levels of other transcriptional drivers or markers of EMT, such as Zeb1, Slug, or Vimentin (Supplementary Fig. S5). HA-tagged MARCH2 coprecipitates with FLAG-SNAIL, supporting a physical interaction between these two molecules (Fig. 4B). Overexpression of WT MARCH2, but not RING domain–mutant (W97A) or C-terminal domain–mutant MARCH2 (35), increased ubiquitination of immunoprecipitated FLAG-SNAIL (Fig. 4C). In *in vitro* ubiquitination assays, recombinant SNAIL is polyubiquitinated in the presence of E1, E2, and MARCH2, further confirming MARCH2’s E3 ligase activity toward SNAIL (Fig. 4D).

**FIGURE 5:**
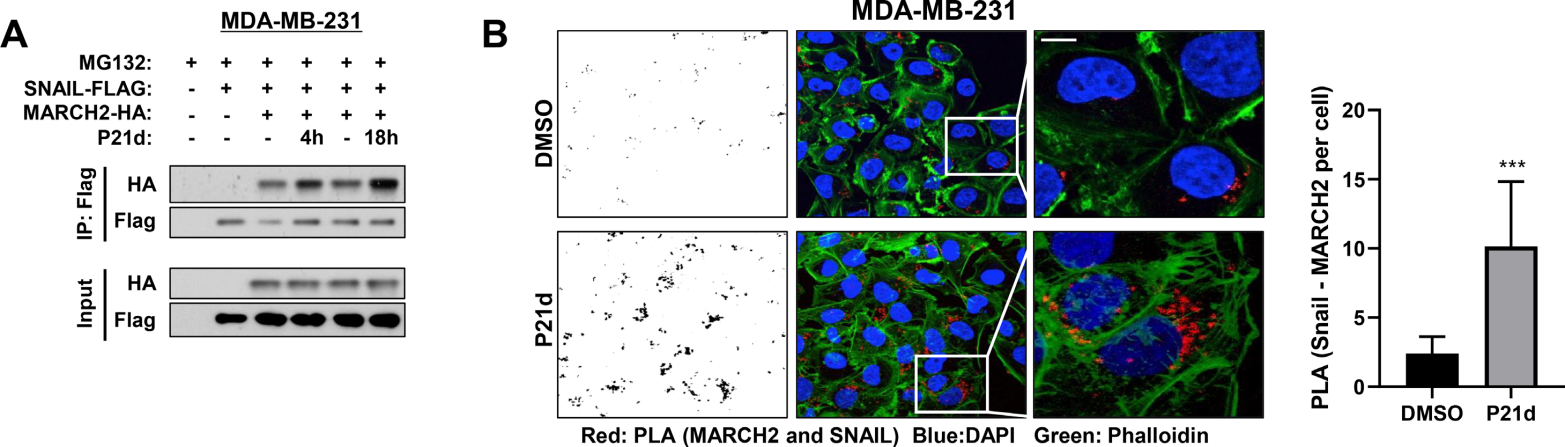
PTK6 inhibition enhances MARCH2:SNAIL association. **A,** MDA-MB-231 cells expressing FLAG-Snail and HA-MARCH2 (WT) were treated with vehicle control or P21d for 4 or 18 hours in the presence of MG132. MARCH2–SNAIL interaction was assessed by immunoblotting FLAG immunoprecipitates with anti-HA antibody. **B,** Endogenous MARCH2–SNAIL interaction following P21d treatment of MDA-MB-231 was assessed by Duolink PLA. Cells were treated with DMSO or P21d for 8 hours and MG132 cotreatment for the last 4 hours. Cells were stained with antibodies to Snail and MARCH2. PLA signals/cell were quantitated using ImageJ (left). Scale bar, 15 µm.

PTK6 inhibition increases the association of MARCH2 with SNAIL. In cells coexpressing FLAG-SNAIL and HA-MARCH2, P21d treatment increased the amount of MARCH2 that coprecipitated with FLAG-SNAIL (Fig. 5A). With P21d treatment, an increase in PLA signals using antibodies for MARCH2 and SNAIL was also observed supporting an increase in association between endogenous SNAIL and endogenous MARCH2 in TNBC cells (Fig. 5B).

Collectively, these data support MARCH2 as a novel SNAIL E3 ligase. The RING domain of MARCH2 is critical for SNAIL ubiquitination and its association with SNAIL is increased by PTK6 inhibition, leading to SNAIL downregulation.

### MARCH2 Suppresses Mesenchymal Phenotype and Metastases of TNBC

**FIGURE 6:**
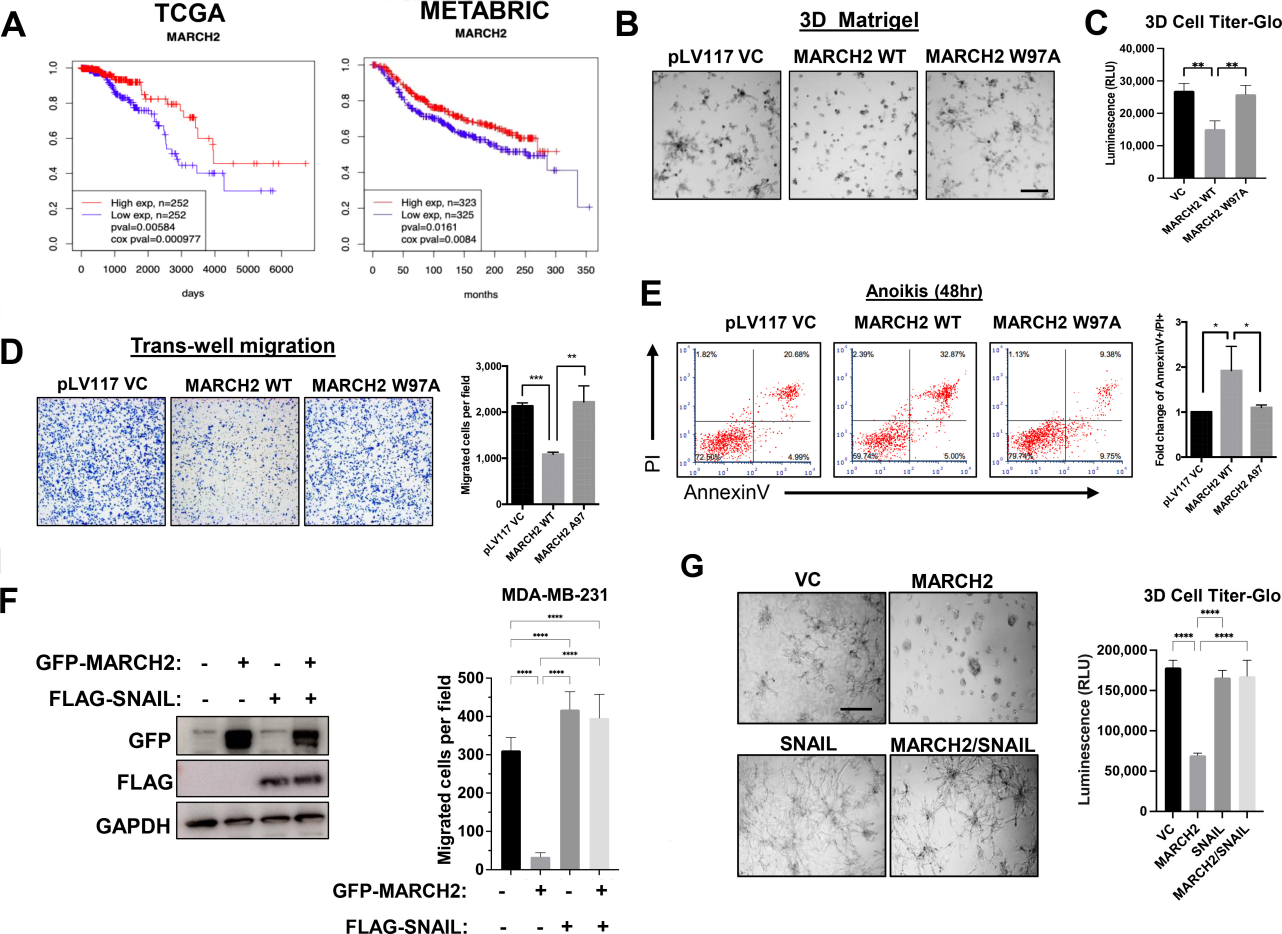
MARCH2 suppresses growth, migration, survival, and metastases of TNBC. **A,** MARCH2 expression is associated with favorable prognosis in patients with breast cancer using TCGA data (*n* = 1,052) and METABRIC data (*n* = 1302). Breast cancer samples were divided into high versus low expression groups based on whether their MARCH2 expression level was higher than upper quartile or lower than lower quartile. **B,** WT, but not RING domain–mutant (W97A), MARCH2 inhibits growth and invasive branching of MDA-MB-231 cells in 3D Matrigel cultures. Representative phase contrast images of 3D cultures of MDA-MB-231 cells overexpressing vector control, WT or RING domain–mutant (W97A) MARCH2 are shown. Scale bars, 30 µm. **C,** Cell Titer-glo 3D was used to measure cell viability of MDA-MB-231 cells expressing control, WT or RING domain–mutant MARCH2 in 3D cultures. Data present mean ± SD. *, *P* = 0.05 by Student *t* test. **D,** Transwell migration assays of MDA-MB-231 cells overexpressing pLv vector control, WT or W97A MARCH2 mutant were counted by ImageJ. Data represent mean ± SEM. **, *P* = 0.01 by Student *t* test. **E,** Anoikis of MDA-MB-231 cells expressing vector control, WT or W97A mutant MARCH2 cultured in suspension for 48 hours was assessed by Annexin/PI staining and flow cytometry. **F,** Coexpression of SNAIL restores migratory capacity of MARCH2-expressing MDA-MB-231 cells. MDA-MB-231 cells coexpressing GFP-MARCH2 and vector control or SNAIL were generated and assessed in transwell assays. Data are representative of three independent experiments each performed with technical triplicates. Data represent mean ± SD. ***, *P* < 0.001 by Student *t* test. **G,** SNAIL restores invasive branching and growth of MARCH2-overexpressing MDA-MB-231 cells in 3D Matrigel cultures. Cell viability was assessed using 3D Cell Titer Glo assays. Data present mean ± SD. ***, *P* < 0.001 by Student *t* test.

In contrast to the poor prognosis associated with higher SNAIL levels, higher levels of MARCH2 are associated with better prognosis in multiple solid tumor types, including breast, pancreatic, endometrial, and head and neck squamous cancers (Fig. 6A; Supplementary Fig. S6). This finding is consistent with MARCH2’s role as a SNAIL E3 ligase that promotes SNAIL degradation, decreasing its levels. In both TCGA and METABRIC datasets, higher expression of MARCH2 in breast cancer was associated with more favorable outcomes.

To determine whether MARCH2 overexpression phenocopies the effects of PTK6 inhibition, as would be anticipated from their concordant effects on SNAIL protein levels, TNBC cells overexpressing MARCH2 were generated and assessed for migration, survival and metastatic capability relative to control. Overexpression of WT MARCH2, but not the RING domain–mutant W97A, increased suppressed growth of MDA-MB-231 cell colonies in 3D culture, as assessed by 3D Cell Titer-glo, with fewer invasive branches compared with vector control or W97A mutant cells (Fig. 6B and C). WT MARCH2 overexpressing MDA-MB-231 cells were less migratory and more susceptible to anoikis in suspension cultures compared to cells expressing vector control or W97A mutant cells (Fig. 6D and E). Growth, migration, and invasive branching were restored in MARCH2-overexpressing cells by coexpression of SNAIL, supporting SNAIL as a critical mediator of MARCH2-dependent cellular effects (Fig. 6F and G).

**FIGURE 7:**
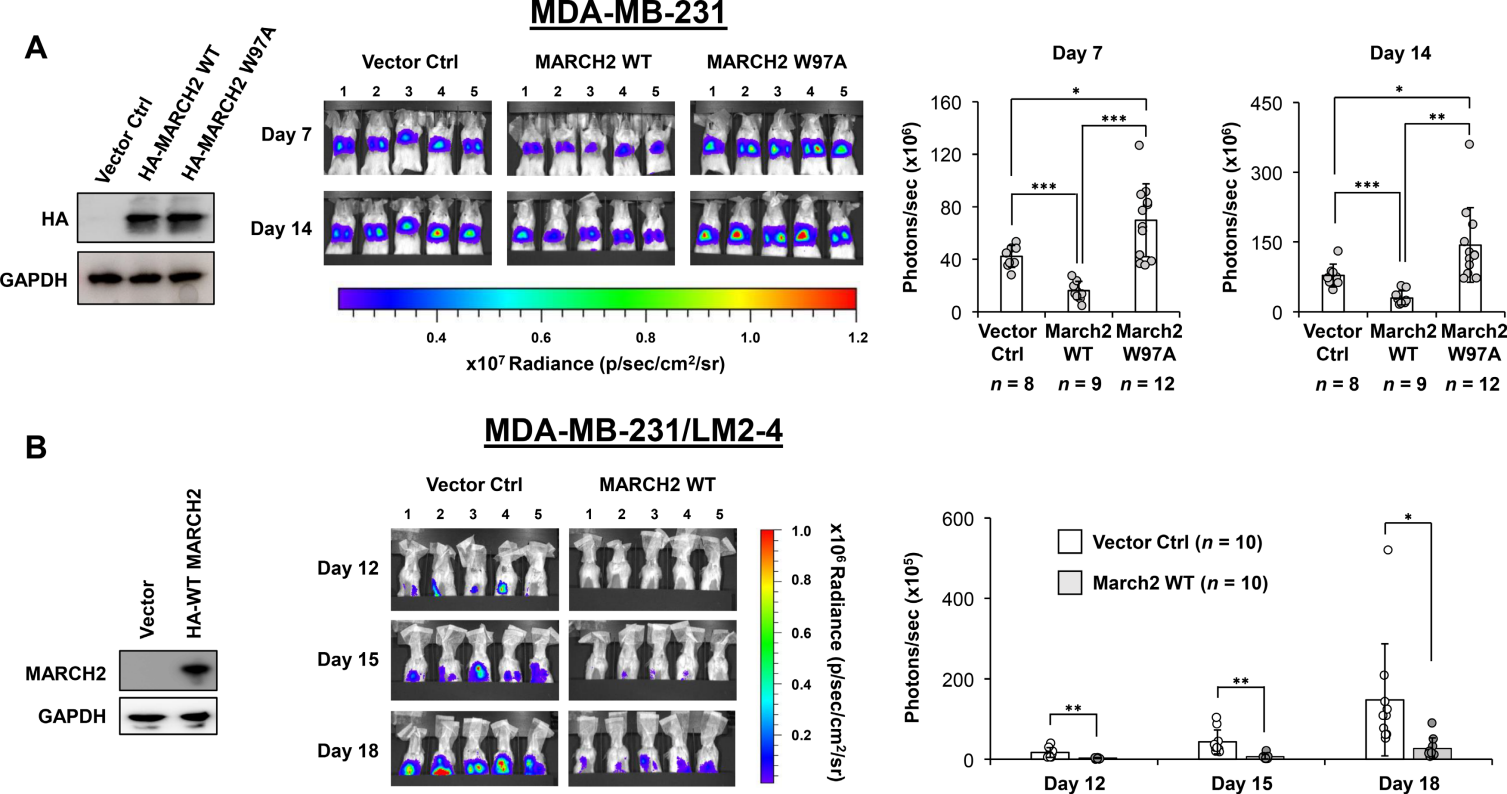
MARCH2 suppresses metastases of TNBC cells. **A,** Metastatic lung colonization by MDA-MB-231/Luciferase cells expressing vector control, WT MARCH2, or RING domain–mutant (W97A) MARCH2 following tail vein injection was serially assessed by IVIS imaging. Total photons/second in a 3.3 × 3.3 cm^2^ area around the lung area of each mouse was used for statistical analysis. *P* values were calculated by Student *t* test, and the *P* value less than 0.05 was regarded as statistically significant (*, *P* < 0.05; **, *P* < 0.01; ***, *P* < 0.001). Data represent mean ± SD. **B,** Spontaneous metastases formation in the lungs by MDA-MB-231/LM2-4/Luciferase cells expressing vector control or WT MARCH2 following implantation into left fourth mammary fat pad implantation was serially assessed by IVIS imaging. Total photons/second in a 3.3 × 3.3 cm^2^ area around the upper body area of each mouse was used for statistical analysis. *P* values were calculated by Student *t* test. Data represent mean ± SD.

Finally, overexpression of WT, but not mutant, MARCH2 significantly suppressed metastatic lung colonization of MDA-MB-231 cells following intravenous tail vein injection, as assessed by IVIS imaging (Fig. 7A). We also assessed the effect of WT MARCH2 expression on the development of spontaneous metastases using MDA-MB-231/LM2-4/Luciferase cells, a highly metastatic variant of MDA-MB-231 cells. Following orthotopic implantation into the mammary fat pad, there was no significant difference in primary tumor growth between vector control and WT MARCH2 expressing tumors (Supplementary Fig. S7). However, MARCH2 overexpression inhibited spontaneous lung metastases as assessed by IVIS imaging (Fig. 7B).

## Discussion

EMT is a process exploited by many cancers that is characterized by changes in adhesion molecules, cell polarity, and acquisition of migratory and invasive properties (1, 2, 4, 39, 40). It is associated with enhanced metastatic capacity and dissemination of tumor cells. EMT has also been implicated in the generation of cancer stem cells that have the capacity to self-renew and are resistant to chemotherapy, targeted therapeutics and immunotherapies (3–6). Therefore, drivers of EMT are attractive candidate therapeutic targets.

PTK6 is a novel EMT driver that promotes metastasis of TNBC. PTK6 stabilizes the expression of SNAIL, a member of a family of zinc finger transcription factors that represses the transcription of E-cadherin (21). SNAIL expression is regulated both transcriptionally and posttranslationally (13–19). TGFβ-activated SMAD 2/3/4 complexes, STAT3 and NFκB, among other transcription factors, directly stimulate SNAIL transcription. Snail transcription has also been shown to be regulated via epigenetic mechanisms such as methylation and acetylation; the dynamic balance between these processes determines the level of Snail transcription (41, 42).

SNAIL is a highly unstable protein and SNAIL protein stability is regulated posttranslationally through phosphorylation, acetylation and ubiquitination. For example, phosphorylation by GSK3β in the SRD of SNAIL promotes its interaction and ubiquitination by β-TRCP, and subsequent proteasome-dependent degradation (16). While some ubiquitin ligases like β-TRCP and FBXO22 are GSK3β dependent, others like FBXL14 and FBXO11 are dependent on alternative kinases like PKD1 (17, 18). PPIL2, SPSB3, and TRIM21 have also been reported to ubiquitinate and degrade SNAIL (43–45). We previously showed that PTK6 inhibition promoted the proteasome-dependent degradation of SNAIL via a mechanism that is independent of GSK3β and known E3 ligases (21). In this report, a screening approach led to the identification of MARCH2 as a novel SNAIL E3 ligase and its association with SNAIL is enhanced by PTK6 inhibition.

MARCH2 is a member of the MARCH family of E3 ligases that consist of 11 members. It is a transmembrane protein with an N-terminal RING C domain that is associated with ubiquitin ligase activity, two transmembrane motifs, and a C-terminal PDZ domain (29–33). MARCH2 is mostly expressed in endosomes, lysosomes, and plasma membrane of cells. Some of the well-known substrates of its E3 ligase activity include DLG1, ADRB2/β2AR, and CFTR (35–37). MARCH2 has been associated with regulation of vesicle trafficking via its interaction with Syntaxin 6 (STX6) and promoting trafficking from the trans-Golgi network to early endosomes (34). MARCH2 was also reported to antagonize Wnt signaling in Xenopus by degrading and downregulating Dsh (46). Interestingly, many of the MARCH family E3 ligases have been implicated in the regulation of immunomodulatory molecules involved in antigen activation or immune cell activation, including MHC class I/II, CD86, and ICAM1 (28, 30, 47). Specifically, MARCH2 downregulates surface expression of the receptor for the inflammatory cytokine IL6 and the integrin α4β1 (48, 49).

There is relatively little known about the role of MARCH2 in the context of tumorigenesis. MARCH2 expression was reported to inhibit autophagy in HeLa cells by promoting ubiquitination and degradation of CFTR (38). Here we have shown that SNAIL is a novel substrate of MARCH2 E3 ligase activity in breast cancer cells, and the MARCH2–SNAIL interaction is enhanced by PTK6 inhibition. MARCH2 overexpression phenocopies the effects of PTK6 inhibition and SNAIL downregulation, inhibiting growth, migration, and metastases. These effects support a tumor suppressive role for MARCH2 expression and is consistent with the better prognosis associated with higher MARCH2 expression in breast and other cancers. Ongoing studies will further clarify the mechanisms by which MARCH2 is regulated by PTK6 in the context of various oncogenic activities in multiple tumor types. It is also of interest to understand how MARCH2 is regulated in other tumor types and its other substrates may be important for its tumor suppressive activity.

The tumor suppressive effects of MARCH2 overexpression in TNBC cells (inhibition of migration, proliferation, and survival) contrast with the recently reported role of MARCH2 in colon cancer cells. MARCH2 downregulation in HCT116 cells inhibited tumor xenograft growth, suggesting a protumorigenic role in this context (50). In our studies, knockdown of MARCH2 in TNBC cells does not significantly affect growth (Supplementary Fig. S8). It is likely that there are context-specific functions of MARCH2 with differential substrates depending on tissue type. Of note, PTK6 also exhibits a contrasting role in colon cancer cells versus other tumor types; PTK6 knockdown in SW480 and HCT116 cells promotes EMT and increased growth of SW480 tumor xenografts (51). These effects of PTK6 contrast with those observed in TNBC and other cancer cell line models (21, 52–56). Therefore, PTK6- and MARCH2-dependent regulation of EMT may have unique tissue context-specific functions in colonic tissue.

In summary, we have identified a novel mechanism by which oncogenic PTK6 promotes SNAIL stabilization and EMT in TNBC cells. SNAIL adds to the growing list of substrates of MARCH2 E3 ligase activity. It is possible that other established or novel substrates also contribute to MARCH2’s tumor suppressive effects on primary tumor growth and metastases. The potential effects of MARCH2 on tumor growth and metastases in the context of immunocompetence are especially intriguing to consider given its effects on immune signaling and immunoregulatory molecules and are the focus of ongoing studies.

## Supplementary Material

Figure S1 Figure S1 shows Kaplan-Meier curves associated with EMT driver genes

Figure S2 Fig S2 shows the effect of PTK6 kinase inhibitor 4f on SNAIL levels in TNBC cells

Figure S3 Figure S3 shows validation of Proximity Ligation Assay to detect protein ubiquitination

Figure S4 Figure S4 shows validation of screening approach and novel SNAIL E3 ligase candidates identifed by screen

Figure S5 Figure S5 shows effect of MARCH2 on levels of EMT driver proteins

Figure S6 Figure S6 shows prognostic significance of MARCH2 levels in different cancer types

Figure S7 Figure S7 shows effect of MARCH2 overexpression on primary tumor growth of MDA-MB231 LM2-4 primary xenografts

Figure S8 Figure S8 shows effect of MARCH2 shRNA expression on growth of MDA-MB231 cells
