# Supplementary material for: MARCH2, a Novel Oncogene-regulated SNAIL E3 Ligase, Suppresses Triple-negative Breast Cancer Metastases
Source: Cancer Res Commun. 2024 Mar 28;4(3):946–57. doi: 10.1158/2767-9764.CRC-23-0090 (PMC10977041; doi:10.1158/2767-9764.CRC-23-0090)
Supplement: Figure S1 — shows Kaplan-Meier curves associated with EMT driver genes [file crc-23-0090-s01.pdf]

Supplemental Figure 1

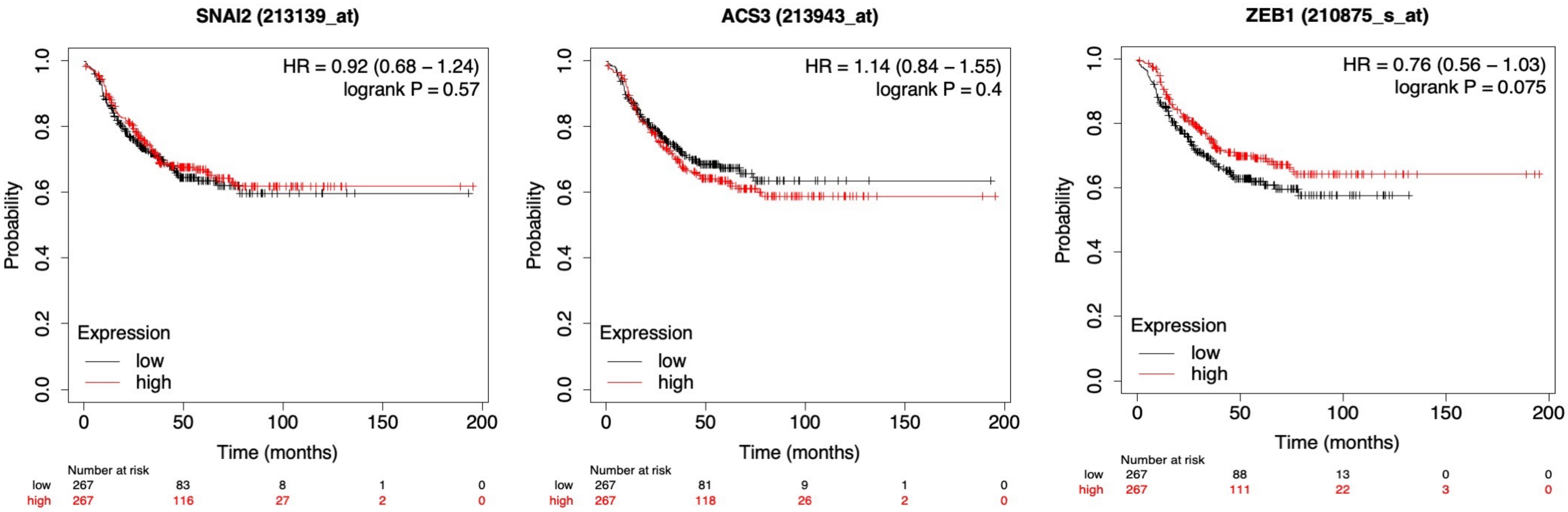

Supplemental Figure 1. Kaplan-Meier curves of EMT drivers in triple negative breast cancer. RNA expression levels of EMT drivers, Snail 2 (Slug), Twist and Zeb1, were divided across the median into high and low expressors, and survival curves were plotted.
