# Supplementary material for: MARCH2, a Novel Oncogene-regulated SNAIL E3 Ligase, Suppresses Triple-negative Breast Cancer Metastases
Source: Cancer Res Commun. 2024 Mar 28;4(3):946–57. doi: 10.1158/2767-9764.CRC-23-0090 (PMC10977041; doi:10.1158/2767-9764.CRC-23-0090)
Supplement: Figure S2 — Fig S2 shows the effect of PTK6 kinase inhibitor 4f on SNAIL levels in TNBC cells [file crc-23-0090-s02.pdf]

Supplemental Fig 2

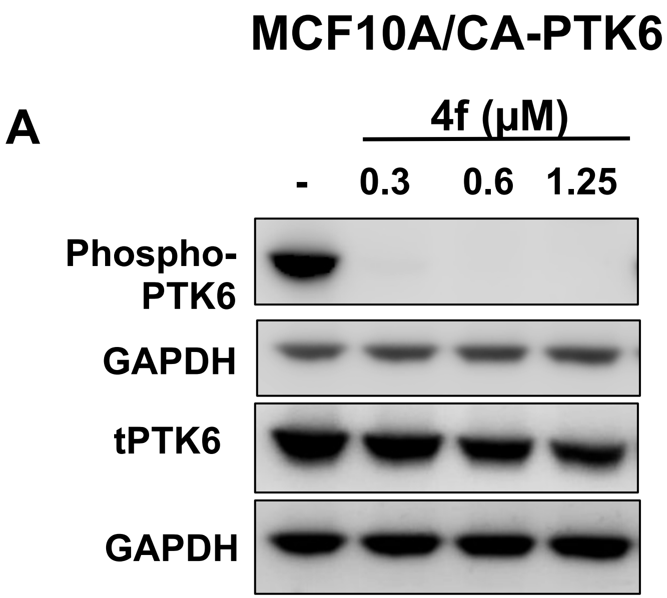

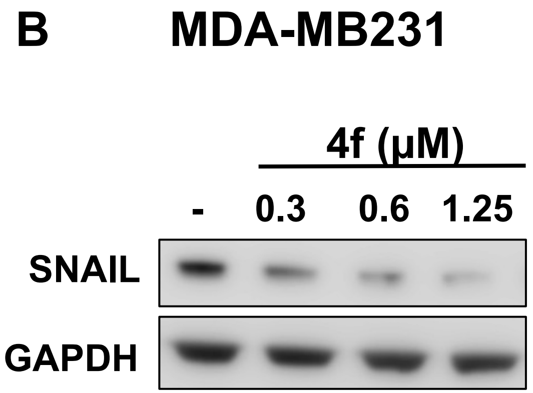

Supplemental Figure 2. Effect of PTK6 kinase inhibitor 4f (Millipore Sigma) on SNAIL levels in TNBC cells

A) Validation of 4f as a PTK6 kinase inhibitor. MCF10A cells overexpressing a constitutively active PTK6 were treated with the indicated concentrations of 4f. Lysates were probed with anti-phosphoPTK6 (tyrosine 342) antibody. B) MDA-MB231 cells were treated with 4f and levels of SNAIL were assessed in lysates.
