# Supplementary material for: MARCH2, a Novel Oncogene-regulated SNAIL E3 Ligase, Suppresses Triple-negative Breast Cancer Metastases
Source: Cancer Res Commun. 2024 Mar 28;4(3):946–57. doi: 10.1158/2767-9764.CRC-23-0090 (PMC10977041; doi:10.1158/2767-9764.CRC-23-0090)
Supplement: Figure S3 — shows validation of Proximity Ligation Assay to detect protein ubiquitination [file crc-23-0090-s03.pdf]

Supplemental Figure 3

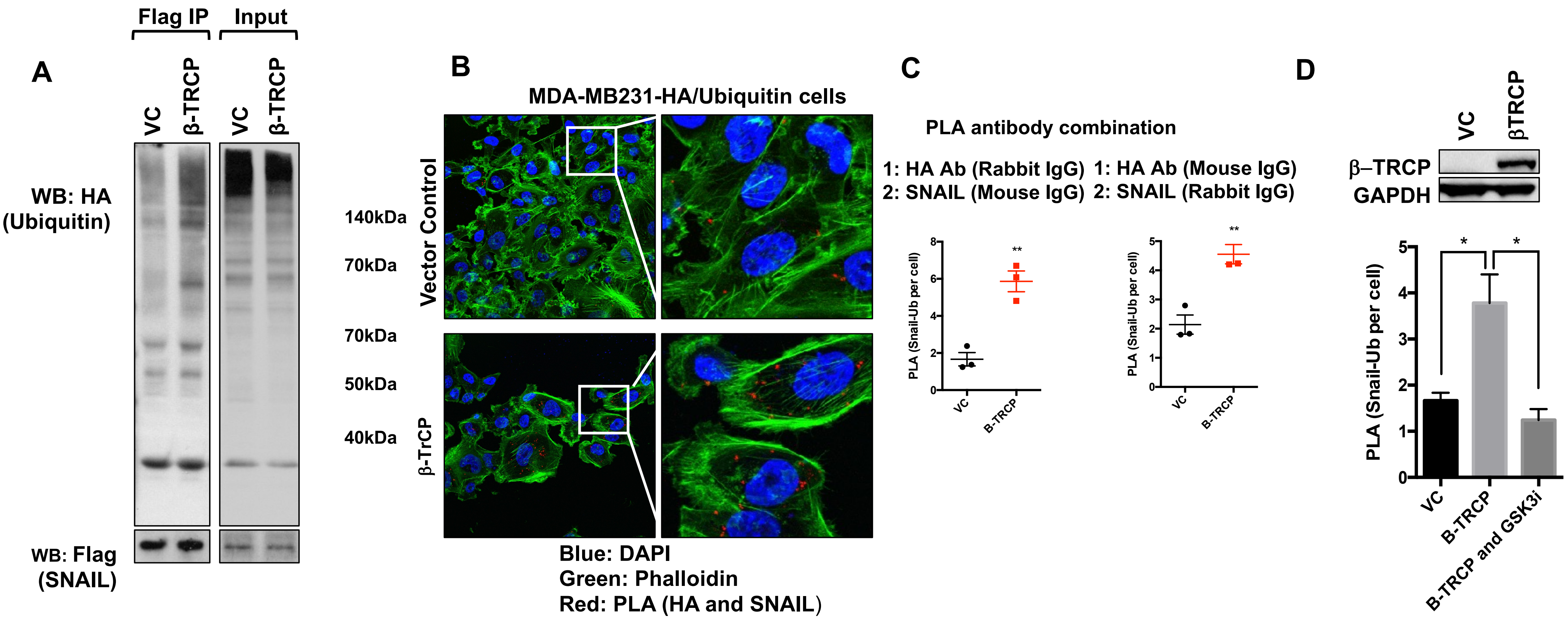

Supplemental Figure 3. Validation of PLA as a method to detect protein ubiquitination. A) β-TRCP, an established SNAIL E3 ligase, promotes ubiquitination of immunoprecipitated FLAG-SNAIL. MDA-MB231 cells co-expressing HA-Ubiquitin, FLAG-SNAIL and vector control or βTRCP were treated with MG132 and lysed. Anti-FLAG antibody was used to immunoprecipitate SNAIL and immunoprecipitates were blotted for Ubiquitin using anti-HA antibody. B) Representative images of proximity ligation assay (PLA) performed on MG132-treated MDA-MB231 cells co-expressing HA-ubiquitin and vector control or βTRCP, using anti-HA (Ubiquitin) and anti-SNAIL antibodies. C) Quantitation of PLA signals/cell using different isotype combinations of anti-HA (Ubiquitin) and anti-SNAIL antibodies. At least 90 cells were counted. D) GSK3β inhibitor (TWS119) treatment of β-TRCP overexpressing cells decreases SNAIL/ubiquitin PLA signals.
