# Supplementary material for: MARCH2, a Novel Oncogene-regulated SNAIL E3 Ligase, Suppresses Triple-negative Breast Cancer Metastases
Source: Cancer Res Commun. 2024 Mar 28;4(3):946–57. doi: 10.1158/2767-9764.CRC-23-0090 (PMC10977041; doi:10.1158/2767-9764.CRC-23-0090)
Supplement: Figure S4 — shows validation of screening approach and novel SNAIL E3 ligase candidates identifed by screen [file crc-23-0090-s04.pdf]

Supplemental Fig 4

A

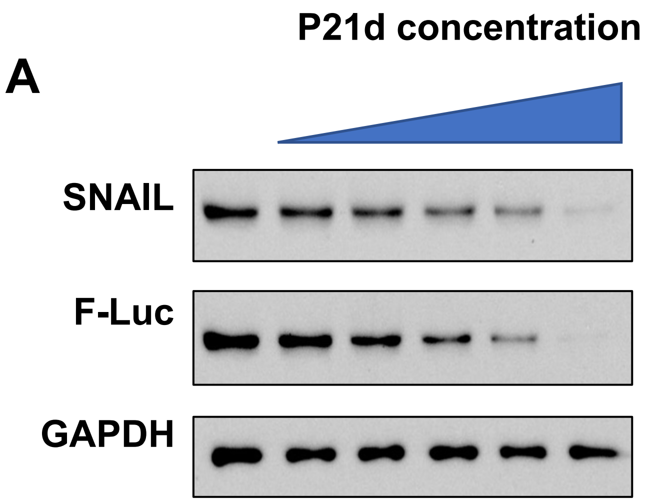

B

Loocation	Gene name	Recovery
5-3 #6	MARCH2	3.790315732
2-6 #6	FBXW8	2.332316654
4-8 #1	RNF5	2.054217547
8-8 #9	NHLRC1	2.052178248
2-3 #9	FBXO18	1.820664834
2-8 #3	FBXO2	1.745462311
3-8 #3	RNF32	1.565603749
2-7 #2	FBXO24	1.526517876
5-3 #2	UBE4B	1.50392579
3-5 #1	NEURL2	1.452411573
4-5 #5	TRIM63	1.353733062
5-6 #1	RNF112	1.297126112
8-1 #2	MNAT1	1.29348143

Supplemental Figure 4. Screen for PTK6-dependent Snail E3 ligases using MDA-MB231 cells overexpressing Snail-F-Luc construct. A) PTK6 kinase inhibitor P21d treatment decreases expression of F-Luc SNAIL, similar to the effects on endogenous SNAIL. Western analysis was performed on lysates of P21d-treated cells using antibodies against SNAIL or F-Luciferase B) Candidate SNAIL E3 ligases identified by primary and secondary screening.
