# Supplementary material for: MARCH2, a Novel Oncogene-regulated SNAIL E3 Ligase, Suppresses Triple-negative Breast Cancer Metastases
Source: Cancer Res Commun. 2024 Mar 28;4(3):946–57. doi: 10.1158/2767-9764.CRC-23-0090 (PMC10977041; doi:10.1158/2767-9764.CRC-23-0090)
Supplement: Figure S5 — shows effect of MARCH2 on levels of EMT driver proteins [file crc-23-0090-s05.pdf]

Supplemental Figure 5

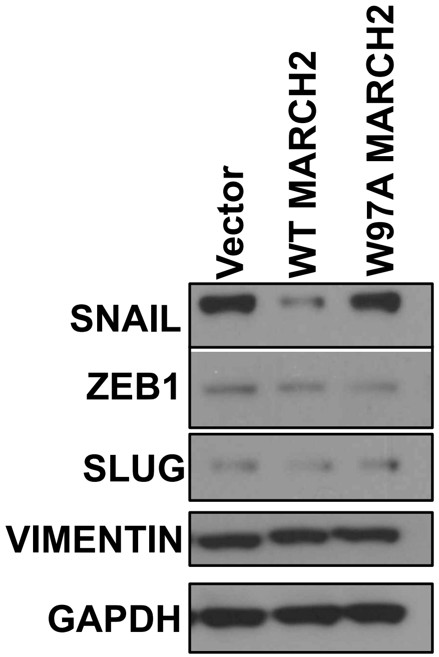

Supplemental Figure 5. MARCH2 does not affect expression of other epithelial-mesenchymal transition (EMT) drivers. Expression of SNAIL, ZEB1 and SLUG were determined in MDA-MB231 cells over-expressing wild type or RING domain mutant MARCH2.
