# Supplementary material for: MARCH2, a Novel Oncogene-regulated SNAIL E3 Ligase, Suppresses Triple-negative Breast Cancer Metastases
Source: Cancer Res Commun. 2024 Mar 28;4(3):946–57. doi: 10.1158/2767-9764.CRC-23-0090 (PMC10977041; doi:10.1158/2767-9764.CRC-23-0090)
Supplement: Figure S6 — shows prognostic significance of MARCH2 levels in different cancer types [file crc-23-0090-s06.pdf]

Supplemental Figure 6

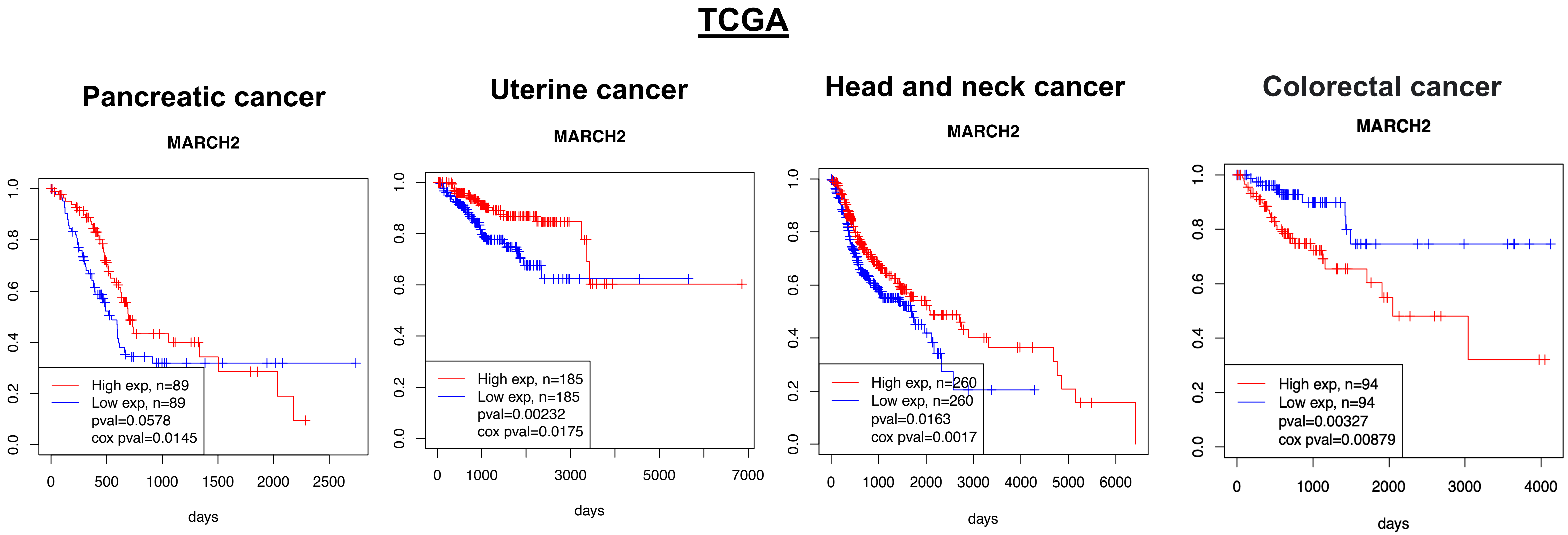

Supplemental Figure 6. Kaplan-Meier (KM) curves of MARCH2 expression in diverse cancer types using TCGA data set. Overall survival is plotted. Cancer samples were divided into high vs low expression groups based on whether their MARCH2 expression level was higher than upper quartile or lower than lower quartile.
