# Supplementary material for: MARCH2, a Novel Oncogene-regulated SNAIL E3 Ligase, Suppresses Triple-negative Breast Cancer Metastases
Source: Cancer Res Commun. 2024 Mar 28;4(3):946–57. doi: 10.1158/2767-9764.CRC-23-0090 (PMC10977041; doi:10.1158/2767-9764.CRC-23-0090)
Supplement: Figure S7 — shows effect of MARCH2 overexpression on primary tumor growth of MDA-MB231 LM2-4 primary xenografts [file crc-23-0090-s07.pdf]

Supplemental Figure 7

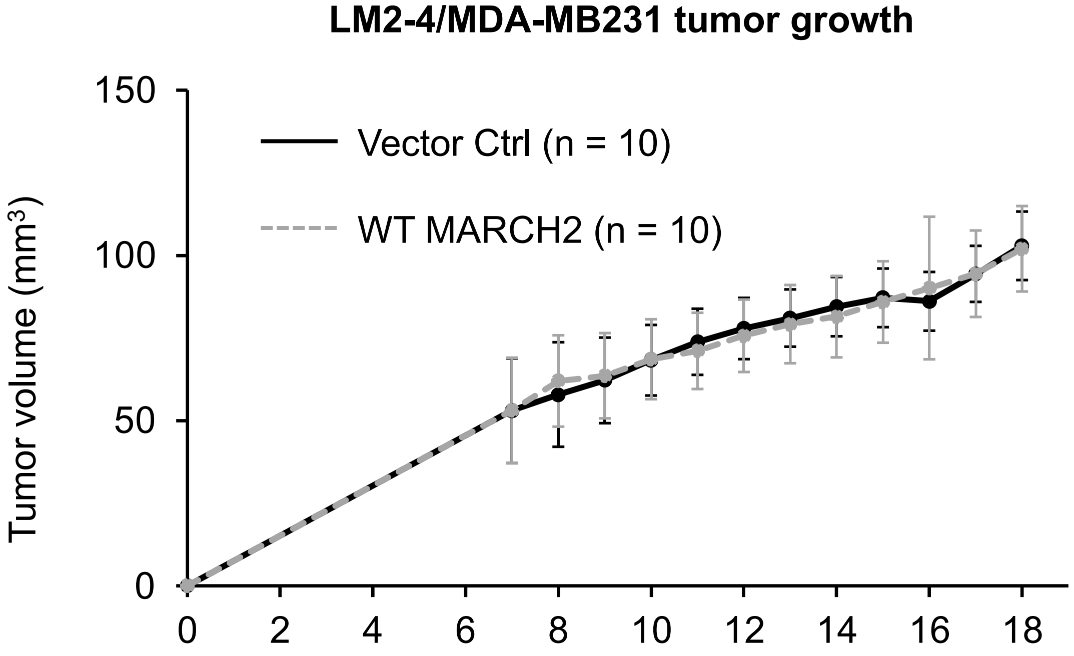

Supplemental Figure 7. Volumes of primary tumors formed following injection of MDA-MB231/LM2-4 Luciferase cells expressing vector control or wild-type MARCH2 into the 4th mammary fat pad implantation were measured on the indicated days. (n = 10 for vector control groups; n = 10 for wild-type MARCH2 groups).
