# Supplementary material for: MARCH2, a Novel Oncogene-regulated SNAIL E3 Ligase, Suppresses Triple-negative Breast Cancer Metastases
Source: Cancer Res Commun. 2024 Mar 28;4(3):946–57. doi: 10.1158/2767-9764.CRC-23-0090 (PMC10977041; doi:10.1158/2767-9764.CRC-23-0090)
Supplement: Figure S8 — shows effect of MARCH2 shRNA expression on growth of MDA-MB231 cells [file crc-23-0090-s08.pdf]

Supplemental Figure 8

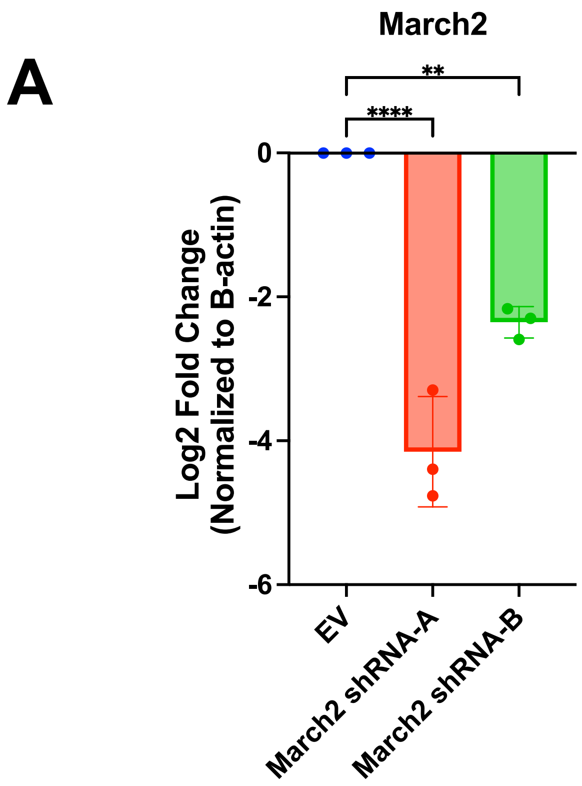

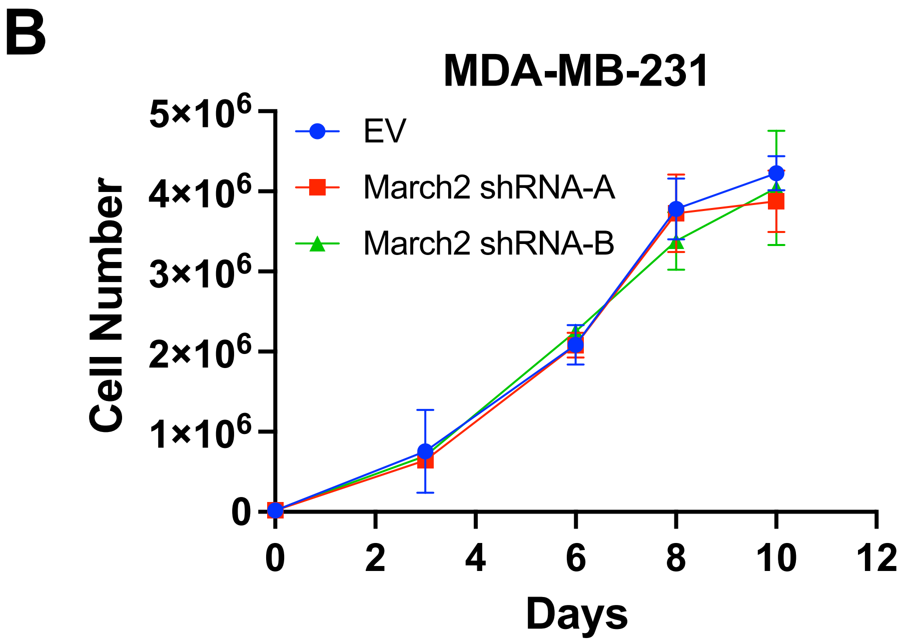

Supplemental Figure 8. MARCH2 shRNA does not affect growth of MDA-MB231 cells.

A) Two independent MARCH2 shRNA vectors that downregulate MARCH2 were identified. qRT-PCR data are shown. B) Growth curves of MDA-MB231 cells expressing empty vector control or MARCH2 shRNA vector are shown.
